# ‘Targeting’ Improved Outcomes with Antibody-Drug Conjugates in Non-Small Cell Lung Cancer—An Updated Review

**DOI:** 10.3390/curroncol30040330

**Published:** 2023-04-20

**Authors:** Saurav Verma, Daniel Breadner, Jacques Raphael

**Affiliations:** 1 Division of Medical Oncology, Department of Oncology, Schulich School of Medicine & Dentistry, Western University, London, ON N6A 5C1, Canada; saurav.verma@lhsc.on.ca (S.V.); daniel.breadner@lhsc.on.ca (D.B.); 2 London Regional Cancer Program, London Health Sciences Centre, London, ON N6A 5W9, Canada

**Keywords:** Antibody-Drug conjugate, ADCs, non-small cell lung cancer (NSCLC)

## Abstract

Antibody-Drug conjugates (ADCs) are a relatively new class of drugs with a promise to improve the outcomes in specific cancers. By delivering the cytotoxic agent to tumor cells expressing specific antigens, ADCs achieve a better therapeutic index and more potency. ADCs have been approved for several hematological and solid malignancies, including breast, urothelial and gastric carcinoma. Recently, trastuzumab deruxtecan (TDXd) was the first ADC approved for previously treated metastatic HER2-mutant non-small cell lung cancer (NSCLC). Many promising ADCs are in the pipeline for clinical development in non-small cell lung cancer, including sacituzumab govitecan, patritumab deruxtecan, datopotamab deruxtecan and tusamitamab ravtansine. There is a hope that these drugs would cater to the unmet need of specific patient populations, including patients with currently untargetable mutations. We hope these drugs, e.g., TROP2 targeted ADCs, will also give more options for therapy in NSCLC to improve outcomes for patients. In this comprehensive review, we will be discussing the recent evidence including targets, efficacy and the safety of newer ADC candidates in NSCLC. We will also briefly discuss the specific toxicities, novel biomarkers, overcoming resistance mechanisms, challenges and the way forward, as these new ADCs and combinations find a way into the clinical practice.

## 1. Introduction

Lung cancer is the second most common cancer worldwide and the leading cause of cancer-related deaths [1]. Non-small cell lung cancer (NSCLC) accounts for approximately 85% of all new lung cancer cases and more than two-thirds are advanced/metastatic at the initial diagnosis. With the continuing advancements in the genomic underpinnings of lung cancer, it is now at the helm of personalized therapy. The current systemic therapeutic arsenal of non-small cell lung cancer comprises chiefly immune checkpoint inhibitors (ICIs), targeted therapy and chemotherapy. Although these therapies have significantly improved outcomes, there is hope for better options for specific populations lacking targetable driver mutations and those with the progression of disease on first-line chemo-immunotherapy, which represent the largest unmet need in advanced/metastatic NSCLC with limited therapeutic options and poor prognosis. 

Antibody-Drug conjugates are a rapidly evolving class of biotherapeutics combining cytotoxic drugs and targeted antibodies. Combining these two moieties with a ‘linker’ delivers a biotherapeutic that can deliver the drug selectively to cells harboring the target (antigen) for antibodies, thereby, increasing safety and consolidating efficacy. There has been a rapid development of ADCs in lung cancer over recent years. Recently, in August 2022, trastuzumab deruxtecan was approved by Food and Drug Administration (FDA) for previously treated human epidermal growth factor receptor 2 (HER2) mutant NSCLC, based on a phase 2 study showing improved outcomes. Besides trying to improve outcomes in patients with specific patient populations with a targetable mutation, trials are evaluating ADCs in combination with ICIs, tyrosine kinase inhibitors (TKIs) and chemotherapy to improve outcomes in first- and subsequent-line settings. The focus has also been to increase therapeutic index/window of ADCs by approaches such as more selective targets, increased drug–antibody ratio (DAR), payload-linker optimization and more potent cytotoxic moieties. This review focuses on ADCs in recent clinical and preclinical development for NSCLC.

## 2. ADCs—Structure and Mechanism of Action

### 2.1. Structure

**Figure 1:**
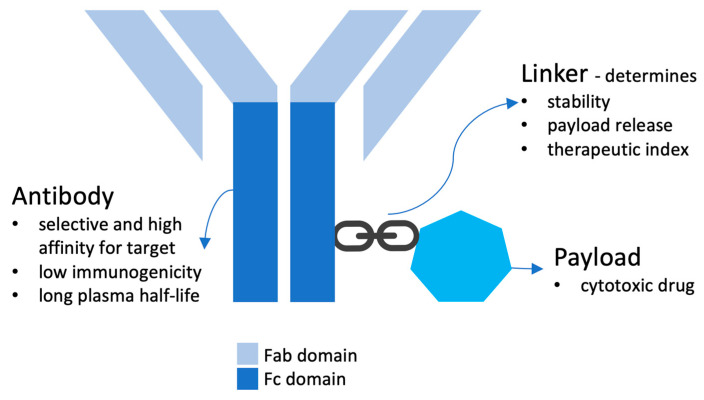
Structure of ADCs.

ADCs consist of a targeted antibody which is attached to a potent cytotoxic agent called ‘payload’ via a chemical ‘linker’ [2] (Figure 1).

#### 2.1.1. Antibody

The antibody, mostly immunoglobulin G, is designed to target a specific antigen or receptor which is usually highly expressed on the target/cancer cell. This selectivity for the antigen on cancer cells is what makes the ADCs highly selective to tumors and thereby minimizing systemic exposures. The binding affinity between the antibody and the surface antigen defines the efficiency of internalization. With high binding affinity to the target antigen and efficient internalization, an ideal antibody moiety should also exhibit low immunogenicity and a long plasma half-life [3]. Currently, fully humanized antibodies with significantly reduced immunogenicity are increasingly used compared to murine and chimeric antibodies.

#### 2.1.2. Payload

Potent cytotoxic agents such as tubulin inhibitors, DNA damaging agents and immunomodulators are the ‘payloads’ of ADC. These possess favorable physicochemical properties, including acceptable hydrophilic/hydrophobic balance, cellular permeability and good stability [4]. Presently, the majority of ADCs use the auristatins (i.e., monomethyl auristatin E (MMAE), monomethyl auristatin F (MMAF)), maytansines (e.g., DM1, DM4), calicheamicins and duocarmycin derivatives.

#### 2.1.3. Linker

The linker binds the antibody to the cytotoxic payload. It defines several characteristics of ADC, such as stability, payload release and therapeutic index. An ideal linker should not induce ADC aggregation, and it is expected to limit premature release of payloads in plasma [2]. These linkers can be cleavable or non-cleavable. The conjugation method, such as stochastic and site-specific conjugation, which connects the linker and payload to the antibody also modulates the characteristics of an ADC.

### 2.2. Mechanism of Action

**Figure 2:**
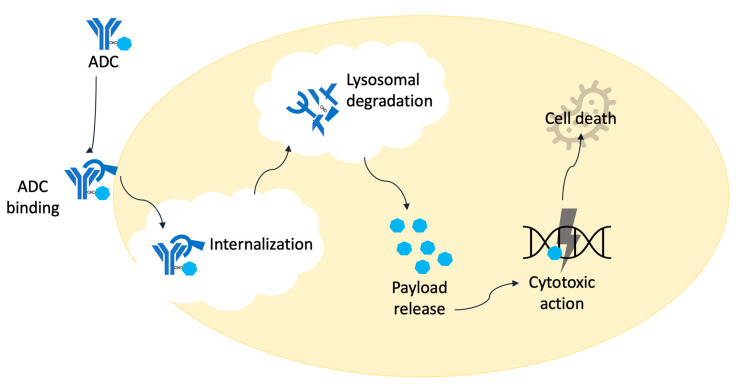
Mechanism of action of ADCs.

The ideal ADC selectively finds out its target, destroys it and has minimal ‘off target’ effects. The ADCs’ mechanism of action display antibody-mediated receptor binding on the surface of the cancer cell. This is followed by internalization of the ADC through receptor-mediated endocytosis with the formation of a clathrin-coated early endosome containing the ADC–antigen complex. Inside the lysosome, degradation of ADC occurs by lysosomal cleavage. This results in the release of free potent payload leading to apoptosis or cell death depending on the cytotoxic mechanism of payload [5] (Figure 2). Various ADCs differ based on the type of target antigens, antibody, payload, linker and conjugation platform (Table 1). 

The ‘bystander effect’ is a specific characteristic of an ADC. This involves either the release of payload from the target tumor or from extracellular space in the vicinity of target cells. The drug is then taken up by the bystander cells which may lack the target. This enhances the killing efficiency of ADCs as the target-negative cells are also killed thereby extending efficacy to heterogenous tumors or cancers with homogenous but low target expression [28,29]. This potent bystander effect is observed with trastuzumab deruxtecan due to its highly membrane-permeable payload, and explains its efficacy in treating tumors with HER2 heterogeneity, resulting in approval in patients with previously treated unresectable or metastatic ‘HER2-low’ breast cancer [30,31]. 

The ADCs have evolved over time from the first generation, characterized by chimeric antibodies, unstable linkers, low/uncontrolled DARs and high immunogenicity to the present fully humanized, stable, highly potent, third-generation ADCs having better conjugation methods yielding better efficacy and therapeutic windows.

### 2.3. Antigenic Targets

The selection of target antigens is the first step in the development of an ADC. The target antigen should be exclusively or predominantly present on tumor cells, preferably extracellular, internalizable and non-secretory. The target antigen determines the mechanism (e.g., endocytosis) for the delivery of cytotoxic payloads into cancer cells. HER2 is a target antigen which is more expressed in tumor cells than normal cells by the order of 10^2^. The various target antigens for ADCs in NSCLC are highlighted in Table 1.

## 3. ADCs in NSCLC

There are several ADCs being studied in NSCLC (Table 2). Various strategies are being used, including combination with chemotherapy or ICIs in various lines of treatment (Table 3).

### 3.1. Trastuzumab Deruxtecan

Trastuzumab deruxtecan (TDXd; DS-8201) is an ADC consisting of a humanized anti-HER2 monoclonal antibody (trastuzumab) linked to a topoisomerase I inhibitor payload (Deruxtecan or DX-8951, an exatecan derivative) by an enzymatically cleavable tetrapeptide-based linker [6]. Despite being conjugated to eight molecules of a potent cytotoxic payload, it has high stability in plasma with a steady delivery. The preclinical studies also showed that it has a highly membrane-permeable payload with a potent bystander effect, which may be beneficial in treating HER2 heterogenous tumors [30]. It has been previously approved for metastatic HER2-positive breast and gastric cancer based on DESTINY-Breast01 and DESTINY-Gastric01 trials [74,75]. 

A phase-I study showed good antitumor activity in HER-2 mutant NSCLC and objective response in 72.7% of the patients (n = 11; 95% confidence interval [CI], 39.0 to 94.0), but interstitial lung disease (ILD) emerged as a specific safety signal [76]. Then, the DESTINY-Lung01, an open-label phase 2 trial, used T-DXd at a dose of 6.4 mg/kg in treatment-refractory HER2-OE or HER2-mutant NSCLC. In the cohort of 91 patients with mutant HER2, the objective response rate (ORR) was 55% (95% CI, 44 to 65). The median duration of response was 9.3 months (95% CI, 5.7 to 14.7). The median progression-free survival and overall survival were 8.2 months (95% CI, 6.0 to 11.9) and 17.8 months (95% CI, 13.8 to 22.1), respectively [34,35]. A recent update at ESMO 2022 reported outcomes on HER2-OE patients, with an ORR of 26.5% and 34.1% at a dose of 6.4 and 5.4 mg/kg, respectively [36]. Based on DESTINY-Lung01, the National Comprehensive Cancer Network (NCCN) granted a category 2A recommendation (preferred over TDM1) for TDXd in previously treated HER2-mutant advanced NSCLC. The increased efficacy of TDXd in HER2-mutant patients compared to HER2-OE patients is explained by preferential internalization of the HER2 receptor ADC complex regardless of HER2 protein expression [77].

The DESTINY-Lung02 evaluated the benefit-risk profile of T-DXd at 5.4 and 6.4 mg/kg in patients with previously treated HER2-mutant metastatic NSCLC. It showed a durable response and clinical activity at both doses. However, the dose of 5.4 mg/kg showed a favorable safety profile [78]. Based on this trial TDXd was approved for patients with metastatic HER2-mutant NSCLC and who have received prior systemic therapy, at a dose of 5.4 mg/kg [79].

Grade 3 or higher adverse events occurred in 46% of patients in HER2 mutant patients in DESTINY-Lung01 trial, with neutropenia (grade 3, 19%) being the most common toxicity. Other common toxicities included nausea, fatigue, alopecia, vomiting, anemia and diarrhea. Notably, drug-related ILD occurred in 26% of patients (N = 24; grade 1 in 3 patients, grade 2 in 15 patients, grade 3 in 4 patients and grade 5 in 2 patients). The drug discontinuation rate following drug-related adverse events was 25%.

In a phase 3 randomized study (DESTINY-Lung04), T-DXd is being tested against the standard of care (investigator’s choice of cisplatin/carboplatin + pembrolizumab + pemetrexed) in patients with advanced/metastatic NSCLC harboring a HER2 exon 19 or 20 mutations [51]. DESTINY-Lung03 is a phase 2 study assessing T-DXd with durvalumab and chemotherapy in advanced/metastatic HER2-OE (immunohistochemistry (IHC) 3+ or IHC 2+) non-squamous (NSQ) NSCLC [52]. The safety of the combination of T-DXd and pembrolizumab in locally advanced/metastatic HER2 positive or HER2 mutant NSCLC is being studied in a phase 1 study [53].

### 3.2. Ado-Trastuzumab Emtansine (TDM1)

T-DM1 is an ADC with anti-HER2 antibody (trastuzumab) linked to emtansine (DM1), an antimicrotubule agent, by a non-cleavable thioether linker. It has a DAR of 3.5.

In a phase-2 study consisting of patients with HER2-OE (IHC 3+ or IHC 2+ with FISH positivity) and HER2 mutations the objective response rate was 6.7% (90% CI: 0.2–32.0). The median follow-up time was 9.2 months. The median PFS and median OS were 2.0 and 10.9 months, respectively. The most common grade 3 or 4 adverse events were thrombocytopenia (40%) and hepatotoxicity (20%). It was concluded that T-DM1 had a limited efficacy for HER2-OE/mutant NSCLC [80]. 

Peters et al. analysed T-DM1 in a phase 2 study with HER2-OE (IHC, 2+ and 3+) and previously treated NSCLC patients and found no treatment responses in IHC 2+ cohort. The ORR was 20% (95% CI, 5.7–43.7%) in IHC 3+ cohort [33]. In a phase II basket trial, Li et al. demonstrated an impressive partial response rate of 44% (95% CI, 22% to 69%) in advanced HER2-mutant lung adenocarcinomas [81]. This led to a category 2A recommendation by the NCCN for use of TDM1 for advanced and previously treated NSCLC with HER-2 mutation. 

Another phase-2 study evaluating TDM1 in previously treated patients with HER2 exon-20 insertion mutations revealed an ORR of 38.1% (90% CI, 23.0–55.9%) and a disease control rate (DCR) of 52.4%. The median duration of response was disappointingly only 3.5 months, and the median progression-free survival and median overall survival were 2.8 and 8.1 months, respectively [32]. Overall, owing to the limited clinical efficacy of TDM1, there are no ongoing phase 3 studies of TDM1 in metastatic NSCLC.

### 3.3. Sacituzumab Govitecan

Sacituzumab govitecan (IMMU-132) is an ADC composed of a Trop-2 IgG1 kappa antibody coupled to SN-38 (a topoisomerase I inhibitor) hydrolysable linker. 

A phase 1/2 IMMU-132-01 basket study (TROPiCS-03) reported clinical activity with sacituzumab govitecan in patients with multiple tumor types not selected for Trop-2 expression including NSCLC. The ORR in the NSCLC cohort was 17% [82]. Heist et al. studied sacituzumab govitecan in patients (N = 54) with pretreated metastatic NSCLC. The ORR was 19%; median response duration, 6.0 months (95% CI, 4.8 to 8.3 months); and the clinical benefit rate 43%. The mPFS was 5.2 months (95% CI, 3.2 to 7.1 months). The grade 3 or higher adverse events included neutropenia (28%), diarrhea (7%), nausea (7%), fatigue (6%) and febrile neutropenia (4%) [46]. Interestingly, more than 90% of 26 assessable archival tumor specimens were highly positive (2+, 3+) for Trop-2 by IHC, and hence any conclusion about the predictive role of Trop-2 could not be made due to the paucity of weakly/negatively stained specimens.

EVOKE-01 is a phase 3 trial evaluating sacituzumab govitecan vs. docetaxel in previously treated advanced or metastatic NSCLC [63]. In EVOKE-02, a phase 2 trial, investigators are studying sacituzumab govitecan and pembrolizumab ± platinum in first-line metastatic NSCLC [45]. In a phase I/II study, MORPHEUS-Lung the combination of atezolizumab with sacituzumab govitecan is being tested for safety and efficacy [66].

### 3.4. Datopotamab Deruxtecan (DS-1062)

Datopotamab Deruxtecan (Dato-DXd) is an ADC consisting of a humanized anti-trophoblast cell surface protein 2 (Trop-2) IgG1 monoclonal antibody linked to a topoisomerase I inhibitor payload (deruxtecan) via a stable tetrapeptide-based cleavable linker. Trop-2 is overexpressed in various epithelial tumors including NSCLC with relatively low expression in normal tissues, and is associated with aggressive tumor behavior [11]. The preclinical studies suggested that Dato-DXd has potential efficacy in Trop-2-positive cancers, and that the anti-tumor activity was proportional to Trop-2 expression.

TROPION-PanTumor01 was a dose-escalation/expansion study evaluating Dato-DXd in patients with advanced NSCLC (N = 175) in the 8 mg/kg vs. 4 and 6 mg/kg cohorts. Based on the results of TROPION-PanTumor01 showing a better tolerance and improved efficacy with a dose of 6 mg/kg, this dose was selected for the TROPION-Lung01 trial, a randomized, phase 3 trial [42]. An updated analysis from a phase 1b study, TROPION-Lung02, in previously treated NSCLC (N = 180), showed that a median follow-up was 11.4 months, an ORR of 26% (in 6 mg/kg cohort) and grade ≥ 3 treatment-emergent adverse events (TEAEs) in 47% of patients across all doses. Drug-related ILD occurred in 4% of patients in a 6 mg/kg cohort [43].

The strategy of combining DATO-DXd with pembrolizumab is being evaluated in TROPION-Lung02, a phase 1b, dose-escalation and expansion study. The median treatment duration was 2.7 months and no drug-related ILD has been seen till now. The grade ≥ 3 TEAEs occurred in 43% of patients. The ORR and DCR were 39% and 82.6%, respectively. The ORR and DCR were 69% and 100%, respectively, in treatment-naïve patients [41]. The combination is being further tested in TROPION-Lung08, a randomized phase 3 trial evaluating Dato-DXD plus pembrolizumab vs. pembrolizumab as a first-line treatment for advanced/metastatic NSCLC [61]. The TROPION-Lung04 is a phase 1 study evaluating the combination of Dato-DXD plus Durvalumab with/without carboplatin as a first-line treatment for advanced/metastatic NSCLC.

In the above trials, the common toxicities seen with DATO-DXd included stomatitis, nausea, alopecia, fatigue and uncommonly ILD. Notably, neutropenia and diarrhea, which may be observed with other TROP2-directed ADCs, were infrequent with Dato-DXd.

There is an unmet need for better outcomes with second- and further-line treatments in previously treated EGFR-mutated locally advanced/metastatic NSCLC. TROPION-Lung01, a phase 3 randomized study, is evaluating Dato-DXD vs. docetaxel in previously treated EGFR-mutated locally advanced/metastatic NSCLC [60].

### 3.5. Patritumab Deruxtecan (HER3-DXd, U3-1402)

HER3-DXd is a novel HER3-directed ADC consisting of fully human anti-HER3 mAb (patritumab) which is covalently linked to a topoisomerase I inhibitor payload (deruxtecan) via a tetrapeptide-based cleavable linker. It shows highly efficient internalization into tumor cells and induces tumor cell apoptosis through DNA damage via released DXd [9]. It is stable and has a high DAR (=8). HER3 is universally expressed in primary NSCLC tumors [83].

In a phase 1 trial, patients with locally advanced or metastatic EGFR-mutated NSCLC with prior EGFR TKI therapy (N = 57) were treated with HER3-DXd 5.6 mg/kg IV Q3W and the ORR was 39% (95% CI, 26.0–52.4%). The median PFS was 8.2 months (95% CI, 4.4–8.3%). Interestingly, antitumor activity of HER3-DXd was observed across various mechanisms of EGFR TKI resistance, including EGFR C797S, MET or HER2 amplification, and BRAF fusion) [39]. The group recently reported safety and efficacy in advanced NSCLC without EGFR mutations previously treated with platinum-based chemotherapy (PBC) with or without immunotherapy (N = 47). The ORR was 28% (95% CI, 16–43%) and median PFS was 5.4 months (95% CI, 3.9–12.7%). The most common grade ≥ 3 toxicities were neutropenia (26%), thrombocytopenia (15%) and fatigue (15%), and drug-related ILD occurred in 4 pts (9% grade 1–2; 0 grade ≥ 3) [40].

HERTHENA-Lung01 is a phase 2 study analyzing HER3-DXd in previously treated metastatic EGFR-mutated NSCLC [59]. In HERTHENA-Lung02, a phase 3 study, it is being tested against chemotherapy in a similar patient population [54]. 

Preclinical studies suggest that although EGFR TKI resistance mechanisms do not lead to alterations in HER3, EGFR inhibition leads to feedback HER3 membrane expression. Therefore, targeting HER3 might add to the increased efficacy of EGFR TKI [84]. A phase 1 study is HER3-DXd and osimertinib combination in the first-line setting of EGFR mutated NSCLC [56].

### 3.6. Tusamitamab Ravtansine (SAR408701)

Tusamitamab ravtansine (SAR408701) is an ADC with an antibody against carcinoembryonic antigen-related cell adhesion molecule-5 (CEACAM5) linked to a cytotoxic maytansinoid (DM4).

In a recent phase 1 study, tusamitamab ravtansine was studied in a previously treated locally advanced/metastatic solid malignant population. A total of 71% patients experienced ≥ 1 TEAE, with most common TEAEs being asthenia, decreased appetite, keratopathy and nausea, with an ORR and DCR being 9.7% and 45.2%, respectively [48].

It is being pitted against docetaxel in previously treated CAECAM5-positive metastatic NSCLC in the phase 3 CARMEN-LC03 trial [67]. A phase 2 study is evaluating the efficacy and safety of tusamitamab ravtansine in non-squamous NSCLC patients with negative or moderate CEACAM5 expression tumors and high circulating CEA [69].

### 3.7. Telisotuzumab Vedotin (Teliso-V/ABBV399)

Teliso-V is an ADC with a c-Met antibody (ABT-700) and MMAE, a microtubule inhibitor. A phase 1/2 M14-239 trial (LUMINOSITY) evaluated Teliso-V in previously treated NSCLC patients with c-Met over-expression (OE). c-Met OE was defined ≥ 25% 3+ by IHC (high, ≥50% 3+; intermediate, 25 to <50% 3+) for the NSQ cohort as and for the squamous (SQ) cohort as ≥75% 1+ by IHC. The ORR was 36.5% in the NSQ EGFR wild type (WT) cohort (52.2% in c-Met high group and 24.1% in c-Met intermediate group) but was modest in the NSQ EGFR mutant and SQ cohorts. The most common any-grade AEs were peripheral sensory neuropathy (25.0% any grade, 4% grade ≥ 3), nausea (22.1%) and hypoalbuminemia (20.6%). Ocular side effects included low-grade blurred vision (17% any grade 1/2, 1% grade ≥ 3) and keratitis (13% grade 1/2). Two patients died, including one due to ILD [47]. Based on LUMINOSITY study, U.S. Food and Drug Administration (FDA) granted Breakthrough Therapy Designation (BTD) to Teliso-V for the treatment of patients with advanced/metastatic epidermal growth factor receptor (EGFR) (WT), non-squamous NSCLC with high levels of c-Met-OE whose disease has progressed on or after platinum-based therapy.

A phase 3 study is evaluating Teliso-V vs. docetaxel in previously treated c-Met-OE, EGFR (WT) metastatic non-squamous NSCLC [70]. In another phase 1 study, Teliso-V is being evaluated as monotherapy and in combination with osimertinib or erlotinib or nivolumab in previously treated c-Met-OE/EGFR mutant metastatic NSCLC [71].

### 3.8. Zanidatamab Zovodotin (ZW49)

ZW49 is a novel bispecific/biparatopic (targeting two different non-overlapping epitopes on ERBB2, on extracellular domains 2 and 4) antibody-targeting HER2 and linked to an auristatin toxin with a protease-cleavable linker. In a dose-finding phase 1 study (N = 76), which included patients with NSCLC, ZW49 was found to have a good safety profile with the most common toxicity being keratitis (42%), alopecia (25%), and diarrhea (21%). There were no ILD or treatment related deaths. The confirmed ORR across multiple cancer types was 28% and DCR was 72% [8].

### 3.9. DS-7300

DS-7300 is an ADC directed at B7-H3, with a topoisomerase I inhibitor payload (deruxtecan). A phase I/II study shows that in previously treated unselected patients with B7-H3 expression; the drug was associated with a DCR of 80% [49].

### 3.10. Enopotamab Vedotin (EnaV)

Enapotamab vedotin is an AXL-specific ADC combining AXL-specific IgG1 with the microtubule-disrupting agent MMAE via a cleavable valine-citrulline linker [15]. In a phase 1 study with advanced/metastatic NSCLC, patients EnaV showed an ORR of 19% and a DCR of 50% in EGFR/ALK WT cohort [50].

## 4. Toxicity

Though ‘targeting’ the payload should make the off-target side-effects minimal, toxicities are one of the challenges that are being faced in the development of clinically beneficial ADCs. These may affect the quality of life and can lead to morbidity, treatment discontinuation and sometimes mortality. These toxicities may be because of the target being expressed in normal tissues, or the drug releasing out in the circulation before reaching the target-expressing cancer cells or leaching out of the drug from target cells. Payload-specific toxicities such as fatigue, headache, anemia, neutropenia, thrombocytopenia, increased liver transaminases, nausea, fatigue, peripheral neuropathy and stomatitis are common [85]. However, two toxicities require consideration, pulmonary and ocular toxicity.

Pulmonary toxicity, including ILD, has been reported with several ADCs, especially TDXd, TDM1, HER3-DXd and Dato-DXd. In a pooled analysis of nine studies with TDXd, the incidence of ILD/pneumonitis was 15.4% [86]. The two independent mechanisms involved in drug-induced ILD include direct toxicity due to the payload and indirect, immune-mediated toxicity [87]. Although the precise mechanism is poorly understood, the toxicity appears to be dose-dependent and dose-frequency-dependent. A target-independent uptake of TDXd in pulmonary macrophages followed by the release of free DXd might be associated with the lung toxicity in monkeys [88]. A careful elucidation of the dose is required to get the best risk–benefit ratio with these drugs in future trials. The diagnostic and treatment challenges for ILD are particularly high in pre-treated patients with lung cancer with lung function compromise due to disease, radiation-associated pneumonitis or IO-associated pneumonitis, making clinical decisions tougher. A multidisciplinary approach and careful monitoring to pick up asymptomatic or minimally symptomatic ILD/pneumonitis may allow transient interruption with a rechallenge [89]. However, a more severe form may add to morbidity, lead to cessation of ADC and rarely mortality. 

Ocular adverse effects (AEs) include loss of vision, blurred vision, dry eye, corneal ulcers and microcyst-like epithelial changes (MECs). Mechanism of corneal epithelial toxicity may depend on the specific ADC and may be either an on-target effect (HER2 is expressed in corneal cells) or an off-target toxicity by ‘micropinocytosis-mediated internalization’ of the drug or its metabolite [90]. Dry eye and conjunctivitis have been reported with TDXd and TDM1. The keratopathy appears to be reversible with treatment cessation, though long-term data is still evolving. It has been seen with Tusamitamab ravtansine and Zanidatamab Zovodotin. Careful ocular monitoring is required in patients taking this drug with a transient interruption for grade 1 or superficial punctate grade 2 keratitis, and discontinuation for more severe forms. 

There is a lack of data regarding the concurrent administration of ADCs with radiation in NSCLC. The concern is the possible increase in the incidence of pneumonitis with ADCs given concurrently with chest radiation. In the KATHERINE study on breast cancer, adjuvant local radiation was given concurrently with TDM1 and trastuzumab. Radiation pneumonitis was slightly more common with T-DM1 (1.5%) than with trastuzumab (0.7%), and radiation skin injury was similar (27.6% vs. 25.4%) [91]. Based on this data the administration of TDM1 with concurrent local radiation is considered safe for breast cancer. In a retrospective study with 12 patients with breast cancer and brain metastases, radiation necrosis was observed in 50% of patients who received TDM1 concurrently with radiation vs. 28.6% in those with sequential treatment [92]. However, such safety data is scarce in advanced NSCLC. In the DESTINY-Lung01 trial, a washout period of ≥4 weeks after radiation therapy including palliative stereotactic radiation to the chest was required before enrollment. The washout period was ≥2 weeks if palliative stereotactic radiation was given to any other area. Palliative radiotherapy was permitted to known metastatic sites if it did not affect the assessment of response or interrupt treatment more than the maximum time specified for dose modification. Until we have more data, the safer approach might be to avoid ADCs and concurrent radiation to the chest.

There is a focus on strategies to improve the therapeutic index and reduce toxicity [93]. Improved conjugation methods, e.g., site-specific conjugation, allow a more defined control of DAR and better stability of the payload due to its conjugation to a more stable site. The advancement in the linker technology which includes chemical trigger, linker–antibody attachment and linker–payload attachment will improve efficacy and decrease toxicity [94]. Antibody modifications such as probody–drug conjugates, and the development of bispecific antibodies targeting two tumor-associated antigens decrease on-target toxicity [95,96]. Other strategies include modification of the structure of the payload, modification of dosage regimens and ‘Inverse Targeting Strategy’ [97].

## 5. Biomarkers

With the development of multiple ADCs in NSCLC, biomarkers present an opportunity to personalize the treatment. These include markers for the presence of antibody targets, as well as markers for response and resistance. The evolving technology of mass spectrometry, liquid biopsy, development of quantitative bioanalytical assays and quantitative proteomics are promising.

Conventionally, quantifying target expression on the cancer cells has been a strategy to screen for potential beneficiaries of a drug; though ironically, they have been imperfect predictive biomarkers, e.g., HER2 [98]. For example, TDXd showed increased efficacy in HER2-mutant patients compared to HER2-overexpressing patients. A better insight into these biological mechanisms of antibody-target dynamics would be needed to select the most suitable patients for each ADC. 

Efforts are underway to develop better assays for quantification of the expression of the target to select patients. For example, an immunohistochemical assay, CEACAM5 IHC 769, is being validated to evaluate CEACAM5 expression in FFPE tissue for patient selection in ongoing phase 2 and 3 clinical trials of tusamitamab ravtansine [99]. Calvo et al. found that the clinical outcomes with ABBV-21 correlated with multiple EGFR pathway status biomarkers [100]. Thyparambil et al. evaluated ‘payload’ biomarkers for Trop-2 ADCs, besides the target receptor, as these may confer sensitivity or resistance to cytotoxic and thereby affect clinical response [101]. 

Circulating tumor DNA (ctDNA) can be a noninvasive method of monitoring longitudinal changes in tumor burden and patients’ mutational profiles, and a useful biomarker to delineate response [102]. The combination strategy of ADCs and ICIs requires biomarkers that can elucidate immune checkpoints, immune responses and tumor microenvironment. Predictive models with preclinical and pharmacokinetics/pharmacodynamics variables and modeling tools are being developed and validated to predict the response to ADCs [103].

## 6. Resistance Mechanisms

As with any therapy, after a response, tumor cells acquire resistance to ADCs. Some patients may have de-novo resistance. The dynamic and complex mechanism of action involving an antibody, linker and the cytotoxic means that resistance mechanisms may involve a decrease in target receptor/antigen expression or a mutation, an increase in drug efflux transporters, decreased internalization of ADC, changes in the intracellular trafficking/processing of ADCs and impaired release of the cytotoxic into the cytosol [104]. 

For example, decreased antigen expression and tumor heterogeneity after treatment may contribute to resistance to TDM1 [105]. TDXd is able to overcome this resistance by its ‘bystander effect’ [30]. Dual antibodies (bispecific ADCs) may also overcome this type of resistance [106]. Recurrent mutations of the DNA repair gene SLX4 could mediate secondary resistance to T-DXd [107]. Caveolae-mediated endocytosis resulting in less efficient ADC processing is another novel mechanism of resistance to TDM1 [108]. The strategy of co-treatment with irreversible pan-HER inhibitors, and ADC switching to T-DXd achieved durable responses in a patient with lung cancer and corresponding xenograft model developing resistance to T-DM1 [109]. Until now the ‘component switch strategy’ of finding better targets, antibodies, potent payloads and conjugation mechanisms have been the conventional way of finding improved ADCs. As we better understand the unique resistance mechanisms of various ADCs, we may be able to realize the full potential of these drugs. The development of novel ADCs as well as the strategy of combining ADCs with other chemotherapeutics, targeted therapies or ICIs can lead to improved efficacy. Multiple clinical trials are ongoing to investigate the safety and efficacy of this synergistic combination of therapies. 

## 7. Future

With the advancement in ADC engineering, progress is being made with ‘next generation ADCs’ in achieving more potent payloads, better selectivity of targets, novel, cleavable and stable linkers, high DARs and a better bystander effect. The novel ADCs include bispecific ADCs, dual-payload ADCs, radionuclide ADC and ADCs with immune-stimulating payloads [110,111]. Strategies such as biparatopic antibodies, i.e., direct targeting of two tumour antigens or two different epitopes on the same antigen, can lead to bipratotic ADCs with efficacy in heterogenous tumors [112,113]. The novel conjugation methods, ‘site-specific conjugation’, improve the therapeutic index and exhibits superior pharmacology and safety [114]. One such method involves developing reactive cysteine residues at specific sites in antibodies (yielding THIOMABs), allowing drugs to be conjugated (THIOMAB–drug conjugates) with defined stoichiometry [115].

The paradigm of the target for ADCs is evolving beyond the antigens being expressed in the tumor. New ADCs are focusing on engineered antibodies, ‘Probody’, which are activated by proteolytic cleavage in the tumor microenvironment (TME), thus targeting TME and achieving better precision [116,117].

There are preclinical and clinical studies evaluating the approach of combining ICIs, targeted therapy and chemotherapy with ADCs. The rationale behind the approach is that besides achieving direct cell death by the payload cytotoxic, ADCs exert an immunological response against tumor by engaging immune cells affecting antitumor immunity [118]. Novel combination strategies such as cooperative targeting with AXL-107-MMAE and MAPK pathway inhibitors to target distinct and resistant populations in heterogeneous tumors are encouraging [119].

## 8. Conclusions

The advancements in biochemical engineering coupled with a better insight into the biological underpinnings NSCLC is enabling the discovery of novel targets and ADCs. Trastuzumab deruxtecan has been added to the therapeutic arsenal of previously treated HER2-mutated NSCLC. There are several ADCs in clinical development with encouraging preliminary data. These are new drugs in the paradigm of precision therapy with specific challenges and toxicity. They hold a promise of improving outcomes and may become a standard of care in the near future.

## Figures and Tables

**Table 1:** Antibody-Drug conjugates (ADCs) in non-small cell lung cancer, their targets and components.

ADCs in Clinical Development					
ADC	Target	Antibody	Linker	Payload	DAR
Trastuzumab Deruxtecan (T-DXd) [6]	HER2	Trastuzumab	Cleavable	Deruxtecan	8
Ado-trastuzumab Emtansine (T-DM1) [7]		Trastuzumab	Non-cleavable	Emtansine (DM1)	3.5
Zanidatamab Zovodotin (ZW49) [8]		ZW25	Cleavable	Novel Auristatin toxin	2
Patritumab Deruxtecan (HER3-DXd) [9]	HER3	Patritumab	Cleavable	Deruxtecan	8
Sacituzumab Govitecan (SG) [10]	Trop-2	Sacituzumab	Cleavable	SN-38	7.6
Datopotamab Deruxtecan (Dato-DXd) [11]		Datopotamab	Cleavable	Deruxtecan	4
Telisotuzumab Vedotin (Teliso-V) [12]	MET	ABT-700	Cleavable	Monomethyl auristatin E (MMAE)	3.1
Tusamitamab ravtansine [13]	CEACAM5	Anti-CEACAM5	Cleavable	DM4	3.8
DS-7300 [14]	B7-H3			Deruxtecan	4
Enapotamab vedotin (EnaV) [15]	AXL	AXL-specific IgG1 kappa	Cleavable	MMAE	4
Brentuximab vedotin [16]	CD30	IgG1 kappa	Cleavable	MMAE	4
Enfortumab vedotin [17,18]	Nectin-4	IgG1 kappa	Cleavable	MMAE	3.8
Anetumab ravtansine (BAY 94-9343) [19]	Mesothelin	IgG1 lambda	reducible SPDB linker	DM4	3.2
**ADCs in preclinical development**					
**ADC**	**Target**		**Payload**		
REGN5093-M114 [20]	MET		maytansinoid payload		
XB002 [21]	Tissue Factor		Zymelink Auristatin		
LY3076226	Fibroblast growth factor receptor 3 (FGFR3)		DM4		
ABBV-221 [22]	EGFR		MMAE		
AVID100 [23]	EGFR		DM1		
MGC018 [24]	B7-H3		Duocarmycin		
SGN-STNV [25]	STn		MMAE		
DLYE5953A [26]	LYSE		MMAE		
SAR566658 [27]	CA6		DM4		

**Table 2:** Current Clinical Data on use of Antibody Drug Conjugates (ADC’s) in Non-Small Cell Lung Cancer (NSCLC).

ADC	Study (Phase)	Sample Size (n)	Population	Intervention	Overall Response Rate (ORR)	Disease Control Rate	Median Progression-Free Survival in Months (mPFS) (95% CI)	Median Overall Survival in Months (mOS) (95% CI)	Median Duration of Response in Months (mDOR) (95% CI)	Common Grade Adverse Events (%)
HER-2 or ERBB2										
Ado-Trastuzumab Emtansine/TDM-1	Iwama et al., 2021 Phase 2 [32]	22	Previously treated HER2 exon-20 insertion mutations	T-DM1 (3.6 mg/kg) intravenously every 21 days	38.1% (23.0–55.9)	52.4% (35.2–69%)	2.8 (1.4–4.4)	8.1 (3.5–13.2)	3.5 (2.7–6.5)	Thrombocytopenia (63.6%) Transaminitis (AST—45.5%, ALT—40.9%) ILD (13.6%, no grade ¾)
	Peters et al., 2019 [33]	49 (29, IHC 2+, 20 IHC 3+)	Previously treated HER2-overexpressing (OE) advanced NSCLC	T-DM1 (3.6 mg/kg) intravenously every 21 days	IHC 2+, 0%; IHC 3+, 20% (5.7–43.7)	IHC 3+: 40%	IHC 2+, 2.6 (1.4–2.8); IHC 3+, 2.7 (1.4–8.3)	IHC 2+, 12.2 (3.8–23.3); IHC 3+, 15.3 (4.1-NE)	-	Hypersensitivity Peripheral neuropathy Thrombocytopenia Hepatotoxicity
ZW49	Jhaveri et al. [8], 2022	1	Previously treated locally advanced/metastatic solid malignant tumors	ZW49	28%	72%	-	-	-	Keratitis (42%) Alopecia (25%) Diarrhea (21%)
Trastuzumab Deruxtecan/T-DXd/DS-8201	Li et al., 2022 DESTINY-Lung01 Phase 2 [34,35]	91	Previously treated HER2 mutant NSCLC	T-DXd (6.4 mg/kg) intravenously every 21 days	54.9% (44.2–65.4)	92% (85–97)	8.2 (6–11.9)	18.6 (13.8–25.8)	10.6 (5.8–17.7)	Nausea (73%) Fatigue (53%) Neutropenia (35%) Anemia (33%) Diarrhoea (32%)
	Smit et al., 2022 DESTINY-Lung01 [36]	Cohort 1—49 patients Cohort 1a—41 patients	Previously treated HER2-OE NSCLC	Cohort 1—T-DXd 6.4 mg/kg Cohort 1a—T-DXd 5.4 mg/kg	Cohort 1—26.5% (15–41.1) Cohort 1a—34.1 (20.1–50.6)	Cohort 1—69.4% (54.6-81.8) Cohort 1a—78.0% (62.4–89.4)	-	-	Cohort 1—5.8 (4.3-NE); Cohort 2—6.2 (4.2–9.8)	Cohort 1 vs. 1a Nausea (59.2% and 73.2%) Decreased appetite (44.9% and 46.3%) Fatigue (32.7% and 51.2%) ILD (20.4–4.9%)
	DESTINY-Lung02 2022 NCT04644237 [37,38]	101 (6.4 mg/kg arm) 50 (5.4 mg/kg arm)	Previously treated HER2 mutant NSCLC	T-DXd (6.4 vs. 5.4 mg/kg) intravenously every 21 days	6.4 mg/kg: 42.9% (24.5–62.8); 5.4 mg/kg: 53.8% (39.5–67.8)	6.4 mg/kg: 92.9% (76.5–99.1); 5.4 mg/kg: 90.4% (79–96.8)	-	-	6.4 mg/kg: 5.9 (2.8-NE); 5.4 mg/kg: NE (4.2-NE)	-
HER-3										
Patrizumab Deruxtecan/HER3-DXd/U3-1402	Janne et al., 2021 Phase 1 [39] NCT03260491	57	Previously treated EGFR inhibitor-resistant, EGFR-mutated (EGFRm) NSCLC cohort	HER3-DXd 5.6 mg/kg IV Q3W	39% (26–52.4)	72% (58.5–83)	8.2 (4.4–8.3)	-	-	Grade ≥ 3 AEs Thrombocytopenia (30%) Neutropenia (19%) Fatigue (14%) ILD (all grade: 7%)
	Steuer et al., 2021 Phase 1 [40] NCT03260491	47	Previously treated EGFR-unmutated (EGFR wild-type (WT)) NSCLC cohort	HER3-DXd 5.6 mg/kg IV Q3W	-	28% (16–43)	5.4 (3.9–12.7)	-	5.7 (3.7–10.7)	Grade ≥ 3 AEs Thrombocytopenia (15%) Neutropenia (26%) Fatigue (15%) ILD (all grade: 9%)
TROP2										
Datopotamab Deruxtecan/Dato-DXd/DS-1062	Levy et al., 2022 TROPION-Lung02 Phase 1b [41]	60	Previously treated (cohort 1-2) and treatment naïve (cohort 3-6) advanced/metastatic NSCLC	Cohort 1-2: Doublet (Dato-DXd + Pembrolizumab) Cohort 3-6: Triplet (Dato-DXd + Pembrolizumab + Platinum)	39% Doublet—62% Triplet—50%	82.6% Doublet—100% Triplet—90%	-	-	-	Stomatitis (42%) Nausea (38%) Fatigue (27%)
	Garon et al., 2021 TROPION-PanTumor01 Phase 1 NCT03401385 [42,43,44]	180	Previously treated advanced/metastatic NSCLC	Dato-DXD 8 mg/kg 6 mg/kg 4 mg/kg	24% 26% 24%	-	8.2 (1.5–11.8) mg et al.	-	-	Nausea (52%) Stomatitis (48%) Alopecia (39%) Fatigue (32%) Neutropenia (6%) ILD (11%)
Sacituzumab Govitecan (IMMU-132)	Heist et al., 2017 Phase 1/2 [45,46]	54	Previously treated advanced/metastatic NSCLC	IMMU-132 8 or 10 mg/kg were given on days 1 and 8 of 21-day cycles	19%	68%	5.2 (3.2–7.1)	9.5 (5.9–16.7)	6 (4.8–8.3)	Neutropenia (37%) Diarrhea (61%) Nausea (80%) Fatigue (46%) Pneumonia (13%)
MET										
Telisotuzumab vedotin (Teliso-V)	Camidge et al., 2022 Phase 2 LUMINOSITY (M14-239) [47]	136	Previously treated c-Met–OE advanced/metastatic NSCLC	Teliso-V 1.9 mg/kg IV Q2W NSQ EGFR WT cohort SQ EGFR WT cohort	c-Met OE NSQ EGFR WT—36.5%. 52.2% in c-Met high group	-	-	-	c-Met OE NSQ EGFR WT—6.9 (4.1–NE); c-Met high–6.9 (2.4–NE)	Peripheral sensory neuropathy (25.0%) Nausea (22.1%) Hypoalbuminemia (20.6%)
CEACAM5										
Tusamitamab ravtansine (SAR408701)	Gazzah et al., 2022 Phase 1 [48]	31	Previously treated locally advanced/metastatic solid malignant tumors	Tusamitamab ravtansine ranging from 5 to 150 mg/m^2^	9.7%	45.2%	-	-	-	Asthenia (28%) Decreased appetite (28%) Keratopathy (28%) Nausea (28%)
B7-H3										
DS-7300	Doi et al., 2022 [49]	All cancers–127 SQ NSCLC–5	Previously treated locally advanced/metastatic solid malignant tumors	DS-7300	40%; sq NSCLC	80%; sq NSCLC	-	-	-	Nausea (61%) Infusion-related reaction (35%) Vomiting (31%)
AXL										
Enapotamab vedotin (EnaV)	Ramalingam et al., 2019 [50] Phase 1	26	Previously treated advanced/metastatic NSCLC; EGFR WT/ALK- cohort	EnaV 2.2 mg/kg Q3W	19%	50%	-	-	-	GI toxicities

**Table 3:** Currently active trials assessing Antibody-Drug Conjugates (ADC’s) in Non-Small Cell Lung Cancer (NSCLC).

Drug	Study	Phase	Population	Intervention	Primary Endpoint
HER-2 or ERBB2					
Trastuzumab Deruxtecan/T-DXd/DS-8201	DESTINY-Lung04 NCT05048797 [51]	3	Locally advanced/metastatic NSCLC with HER2 mutation	T-DXd vs. SOC	PFS
	DESTINY-Lung03 NCT04686305 [52]	1	Advanced/metastatic HER2 + NSQ NSCLC	T-DXd and Durvalumab with Chemotherapy	Frequency of AEs and SAE
	NCT04042701 [53]	1	Locally advanced/metastatic HER2+ Breast or HER2+ or HER2m NSCLC	T-DXd with pembrolizumab	DLTs and ORR
HER 3					
Patritumab Deruxtecan/U3-1402	HERTHENA-Lung02 NCT05338970 [54,55]	3	Previously treated advanced/metastatic EGFR-mutated NSCLC	HER3-DXd versus platinum-based chemotherapy	PFS
	NCT04676477 [56]	1	EGFR-mutated advanced/metastatic NSCLC	HER3-DXd with Osimertinib	DLTs
	NCT03260491 [57]	1	Locally advanced/metastatic EGFRm NSCLC progressing after EGFR TKI therapy and ≥1 line of platinum-based chemotherapy	HER3-DXd	Pharmacokinetics, efficacy and safety
	HERTHENA-Lung01 [58,59]	2	Previously treated metastatic EGFR-mutated NSCLC	HER3-DXd	ORR
Trop 2					
Datopotamab Deruxtecan/Dato-DXd/DS-1062	TROPION-LUNG01 NCT04656652 [60]	3	Previously treated EGFR-mutated locally advanced/metastatic NSCLC	Dato-DXD vs. Docetaxel	PFS, OS
	TROPION-LUNG08 NCT05215340 [61]	3	First-line treatment for advanced/metastatic NSCLC	Dato-DXD plus pembrolizumab vs. pembrolizumab	PFS, OS
	TROPION-LUNG04 NCT04612751 [62]	1	Advanced/metastatic NSCLC	Dato-DXD plus Durvalumab with/without carboplatin	Number of Participants with DLTs and Treatment-emergent AEs
Sacituzumab govitecan	EVOKE-01 [63,64] Garassino et al.	3	Previously treated advanced/metastatic NSCLC	SG vs. Docetaxel	OS
	EVOKE 02 [65]	2	Advanced/metastatic NSCLC	SG and Pembrolizumab ± platinum in first-line metastatic NSCLC	ORR, DLTs
	Morpheus Lung [66] NCT03337698	1/2	Previously treated/untreated metastatic NSCLC	Atezolizumab with SG	% Of patients with objective response
CAECAM5					
Tusamitamab ravtansine (SAR408701)	CARMEN-LC03 [67] NCT04154956	3	Previously treated CAECAM5-positive metastatic NSCLC	SAR408701 vs. Docetaxel	PFS, OS
	CARMEN-LC06 [68,69] (NCT05245071)	3	Previously treated patients with negative or moderate CEACAM5-expressing NSQ NSCLC tumors and high circulating CEA levels	SAR408701	ORR
c-MET					
Telisotuzumab vedotin (Teliso-V)/ABBV-399	NCT04928846 [70]	3	Previously treated c-Met OE, EGFR WT metastatic NSQ-NSCLC	Teliso-V vs. Docetaxel	PFS, OS
	NCT02099058 [71]	1	Previously treated c-Met OE/EGFRm metastatic NSCLC	ABBV-399 as monotherapy and in combination with Osimertinib, erlotinib, and nivolumab	Number of patients with AEs and RPTD
EGFR					
ABBV-637	NCT04721015 [72]	1	Relapsed and refractory solid tumors	ABBV-399 as monotherapy and in combination with osimertinib or docetaxel	DLTs, RPTD
Mesothelin					
Anetumab ravtansine	Adjei et al. [73]	1b	Relapsed and refractory solid tumors, Mesothelin expressing	Anetumab ravtansine at 6.5 mg/kg IV Q3W	ORR

## Data Availability

No new data were created or analyzed in this study. Data sharing is not applicable to this article.
